# Differential MMP-14 Targeting by Lumican-Derived Peptides Unraveled by In Silico Approach

**DOI:** 10.3390/cancers13194930

**Published:** 2021-09-30

**Authors:** Jonathan Dauvé, Nicolas Belloy, Romain Rivet, Nicolas Etique, Pierre Nizet, Katarzyna Pietraszek-Gremplewicz, Konstantina Karamanou, Manuel Dauchez, Laurent Ramont, Stéphane Brézillon, Stéphanie Baud

**Affiliations:** 1 P3M, Multi-Scale Molecular Modeling Platform, Université de Reims Champagne Ardenne, 51097 Reims, France; Jonathan.Dauve@ico.unicancer.fr (J.D.); nicolas.belloy@univ-reims.fr (N.B.); manuel.dauchez@univ-reims.fr (M.D.); 2 CNRS UMR 7369, Matrice Extracellulaire et Dynamique Cellulaire (MEDyC), Université de Reims Champagne Ardenne, 51095 Reims, France; romain.rivet@univ-reims.fr (R.R.); nicolas.etique@univ-reims.fr (N.E.); pierre.nizet@univ-reims.fr (P.N.); kon.karamanou@gmail.com (K.K.); lramont@chu-reims.fr (L.R.); stephane.brezillon@univ-reims.fr (S.B.); 3 Department of Cell Pathology, Faculty of Biotechnology, University of Wroclaw, Joliot-Curie 14a, 50-383 Wroclaw, Poland; katarzyna.pietraszek-gremplewicz@uwr.edu.pl; 4 Biochemistry, Biochemical Analysis & Matrix Pathobiology Research Group, Laboratory of Biochemistry, Department of Chemistry, University of Patras, 26110 Patras, Greece; 5 CHU Reims, Service Biochimie Pharmacologie-Toxicologie, 51092 Reims, France

**Keywords:** MMP-14, lumican, in silico approach, molecular docking, dynamics, melanoma

## Abstract

**Simple Summary:**

This work aimed to investigate the interactions of lumican-derived peptides and MMP-14. An in silico approach unraveled key residues in the amino acid sequence of MMP-14 interacting with the Small Leucine-Rich Proteoglycan (SLRP) lumican-derived peptides. The in silico docking analysis demonstrated that the interaction of a cyclic lumican-derived peptide (L9Mc, 12 amino acids) with MMP-14 was preferential with the MT-Loop domain of MMP-14 while the linear lumican-derived peptide (lumcorin, 17 amino acids) interacted more with the catalytic site. L9Mc significantly inhibited the migration of murine B16F1 but not human HT-144 melanoma cells and the activity of MMP-14 but with less efficacy than lumican and lumcorin. This result led us to investigate the effect of L9Mc on cell proliferation, which is independent of MMP-14 activity. L9Mc significantly inhibited the proliferation of B16F1 but not HT-144 melanoma cells in vitro and primary melanoma tumor growth. Altogether, the biological assays validated the prediction of the in silico study.

**Abstract:**

Lumican, a small leucine-rich proteoglycan (SLRP) of the extracellular matrix (ECM), displays anti-tumor properties through its direct interaction with MMP-14. Lumican-derived peptides, such as lumcorin (17 amino acids) or L9M (10 amino acids), are able to inhibit the proteolytic activity of MMP-14 and melanoma progression. This work aimed to visualize the interactions of lumican-derived peptides and MMP-14. Molecular modeling was used to characterize the interactions between lumican-derived peptides, such as lumcorin, L9M, and cyclic L9M (L9Mc, 12 amino acids), and MMP-14. The interaction of L9Mc with MMP-14 was preferential with the MT-Loop domain while lumcorin interacted more with the catalytic site. Key residues in the MMP-14 amino acid sequence were highlighted for the interaction between the inhibitory SLRP-derived peptides and MMP-14. In order to validate the in silico data, MMP-14 activity and migration assays were performed using murine B16F1 and human HT-144 melanoma cells. In contrast to the HT-144 melanoma cell line, L9Mc significantly inhibited the migration of B16F1 cells and the activity of MMP-14 but with less efficacy than lumican and lumcorin. L9Mc significantly inhibited the proliferation of B16F1 but not of HT-144 cells in vitro and primary melanoma tumor growth in vivo. Thus, the site of interaction between the domains of MMP-14 and lumcorin or L9Mc were different, which might explain the differences in the inhibitory effect of MMP-14 activity. Altogether, the biological assays validated the prediction of the in silico study. Possible and feasible improvements include molecular dynamics results.

## 1. Introduction

The extracellular matrix (ECM) is formed of a complex network of macromolecules, such as proteins, glycoproteins, and proteoglycans. This diversity is one of the main characteristics of the ECM. This highly ordered matrix constitutes an architectural support through which cells and, in particular, invasive melanocytes will migrate. ECM is also a reservoir of cellular signals, which can be produced by the surrounding cells or via the degradation of ECM compounds into peptide fragments endowed with biological activity called matrikines [1,2,3].

Matrix metalloproteinases (MMPs) are composed of a family of Zn^2+^-dependent enzymes able to cleave ECM proteins in normal and pathological conditions [4]. MMPs are overexpressed in various human malignancies and contribute to tumor invasion and metastasis by degrading ECM components [4,5,6]. Particularly, MMP-2, MMP-7, MMP-9, and MMP-14 have been associated with tumor invasion and metastasis by their capacity to degrade the ECM [7]. MMP-14, a transmembrane matrix metalloproteinase (MT-MMP), is characterized by a C-terminal domain allowing the anchoring of the protein to the membrane, an MT-Loop being involved in the activation of proMMP-2, and a catalytic domain where a zinc ion is found in the catalytic site, allowing its enzymatic activity. Among the different proteins expressed in the ECM, tissue inhibitors of metalloproteinases (TIMPs) inhibit the activity of MMPs. TIMP-1 was shown to be unable to prevent MMP-14 processing of wild-type progelatinase A in contrast to TIMP-2 and TIMP-3 [8,9]. TIMP-1 is an extremely poor inhibitor of MMP-14 while TIMP-2 and TIMP-3 bind more rapidly to the catalytic domain of MMP-14 than to the one of gelatinase A [10].

Lumican belongs to the family of small leucine-rich proteoglycans (SLRPs) that includes decorin, biglycan, and fibromodulin, among others [11,12]. Lumican is the major keratan sulfate proteoglycan of the corneal stroma [13,14,15,16,17,18], but it is also expressed in other tissues, such as skin [19] and cartilage [20]. Lumican has been identified as a substrate of MMP-14 [7]. Similarly, the degradation of decorin by MMP-14 has been previously described [21]. Only MMP-13 is capable of efficiently cleaving fibromodulin and biglycan [22,23] and has a poor effect on lumican and decorin cleavage [23]. Thus, although they share structural features [24,25], these SLRPs present different affinities for MMP-14. Surface Plasmon Resonance (SPR) binding assays confirmed the direct interaction of the catalytic domain of MMP-14 with lumican with moderate affinity (KD # 275 nM), which leads to complete blocking of the activity. Lumican can act also as a competitive inhibitor [26]. Lumican was shown to protect collagen against MMP-14 proteolysis, leading to inhibition of tumor progression. Despite the fact that lumican binds to MMP-14 with moderate affinity, the strength of this interaction is between the affinity of MMP-14 and its biological inhibitor TIMP-2 (KD ~56.1 nM), which forms a complex with MMP-14 and TIMP-1 (KD ~1530 nM), which does not form a tight-binding complex with MMP-14 [27]. On the other hand, MMP-14 may be considered as a potential cell surface receptor for lumican, since its binding affinity to this metalloproteinase was similar to its binding affinity to the α2 integrin subunit (KD ~200 nM) [28].

The MT-Loop of MMP-14 is a potential exosite target region to develop selective MMP-14 inhibitors [29] and is an interface for molecular interactions that mediate enzyme localization to β1-integrin-rich cell adhesion complexes and regulate MMP-14 functions. In the present study, the role of the MT loop was investigated further to decipher these molecular mechanisms of interaction between MMP-14 and SLRP peptides. 

Modeling their interaction might help in visualizing and obtaining a better understanding of the SLRP substrate specificity of the cleavage by MMP-14 but also in the specific regulation of MMP-14 activity by SLRPs [30,31].

Lumican and its derived peptides were previously shown to decrease melanoma progression [19,32,33]. Lumican was also reported to decrease cell proliferation in osteosarcoma [34], to inhibit cell invasion in prostate cancer [35] and in breast cancer [36,37,38,39,40]. However, lumican was also shown to actually facilitate cancer growth. For instance, lumican was shown to be overexpressed in lung cancer cells and has been implicated in the pathogenesis of tumorigenesis and regulation of cancer cell invasion. The expression level of lumican in cancer cells of lung adenocarcinomas was positively correlated with pleural invasion and larger tumor size [41]. More recently, the downregulation of lumican was demonstrated to extend the doubling time of cells and to delay cell growth [42]. Similarly, a significant association between lumican expression and invasive potential in gastric cancer was observed. Lumican was suggested to represent an independent prognostic factor [43]. In the same manner, lumican knockdown was shown to inhibit proliferation and migration in bladder cancer [44]. It was described to be a potential biomarker in bladder cancer [45]. Lumican expression was also shown to be positively correlated with the differentiation of osteosarcoma [34,46]. In addition, lumican was recently demonstrated to facilitate chondrosarcoma cell growth through an IGF-IR/ERK1/2/p53 signaling cascade [47]. These differences in the effect of lumican are closely related to the heterogeneity of its structure [19]. Lumican’s structure might differ from one tissue to another. It is known that lumican is expressed as a keratan sulfate proteoglycan (KSPG) in cornea and cartilage but as a glycoprotein of 57 kDa in skin. This heterogeneity of the lumican structure is associated with opposite regulation of cell migration. For example, lumican is able to stimulate the migration of keratocytes while it inhibits the migration of melanocytes [19]. Interestingly, the post-translational modifications of lumican are correlated with differential regulation of MMP-14 activity [26]. Lumican (57 kDa) is able to inhibit MMP-14 activity and to protect collagen degradation contributing to attenuation of MMP-14-dependent cancer cell migration and invasion [26].

Our group has demonstrated the inhibitory effect of lumican on melanoma progression [19,30]. In addition, previous work from the laboratory has shown that the anti-tumor effects of lumican on murine and human cell lines are mediated through interaction with the α2β1 integrin receptor [28]. 

In parallel, the team was able to highlight a minimal active sequence of the protein having anti-tumor effects similar to those of the total protein [48]. At the Leucine-Rich Repeat (LRR) 9, a 17-residue sequence, called lumcorin (SSLVELDLSYNKLKNIP), is capable of inhibiting the proliferation and migration of melanoma cells [48]. In addition, lumcorin inhibits the expression and activity of MMP-14 similarly to the glycosylated lumican. The minimal sequence mimicking the lumcorin effect could be reduced to 10 residues, ELDLSYNKLK, to give a peptide named L9M. This peptide presents inhibitory effects on MMP-14 activity in vitro [32].

It was shown that a peptide designed from the C-terminal amino acids of lumican (LumC13 or Lumikine) binds to ALK5/TGFβR1 (type 1 receptor of TGFβ) to promote wound healing [49]. In addition, the mechanism by which this synthetic C-terminal Lumikine binds to ALK5 was evaluated. These authors showed that Lumikine derivatives were able to promote corneal epithelial cell migration and corneal wound healing, respectively. Moreover, a computational approach allowed them to design inhibitory peptides based on the interactions of the receptor and ligands [50].

In the present study, molecular modeling was used in order to characterize, at the atomic scale, the interactions between lumican-derived peptides and MMP-14. Key residues, specific from the interaction between the inhibitory peptides and the MMP-14 protein were identified and our data allowed us to validate a methodology of identification of lumican-derived peptides, which could potentially replicate the effects of biologically known inhibitory peptides on the activity of MMP-14.

## 2. Materials and Methods

### 2.1. Selection of the Investigated Peptides

**Figure 1:**
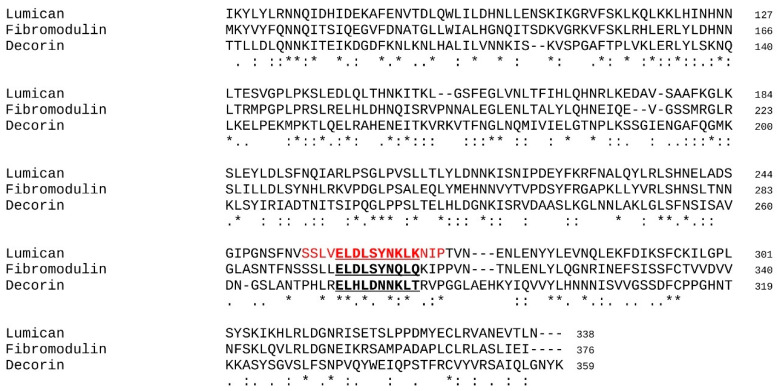
Sequence alignment of lumican, decorin and fibromodulin. The lumcorin peptide is evidenced in red in the lumican amino acid (aa) sequence and the L9M peptide is highlighted in red bold underlined characters. The equivalent D9M and F9M peptides in decorin and fibromodulin aa sequences are highlighted in bold underlined characters, respectively. Alignment ruler: *, identity; homology; similarity.

The peptides derived from lumican used in this study were the following: lumcorin, the peptide corresponding to the LRR9 motif of lumican (SSLVELDLSYNKLKNIP); L9M, the 10 amino acid peptide from lumcorin’s central part (ELDLSYNKLK); L9Mc, the cyclic L9M peptide obtained by the creation of a disulfide bond between additional terminal cysteines; and the scrambled (SCR) peptides (lumcorin SCR—LPSVSILEKLYNNLSKD, L9M SCR—SLELDLNKYK, L9Mc SCR—CSLELDLNKYKC). All peptides were soluble in basal medium. All synthetic peptides were obtained from Genscript (Piscataway, USA). In all experiments, the peptides were used at 100 μM. In addition, peptides derived from two other analogous SLRPs were tested through the in silico protocol designed in this study. The sequence of D9M and F9M was designed based on a sequence alignment performed between human lumican, decorin, and fibromodulin (see Figure 1).

Each 10 amino acid sequence is the equivalent of the LRR9 motif of L9M extracted from lumcorin. Table 1 presents the sequences as well as the known biological associated effects of the peptides used for the in silico investigations and the in vitro and/or in vivo biological experiments.

### 2.2. Design of the In Silico Protocol

**Figure 2:**
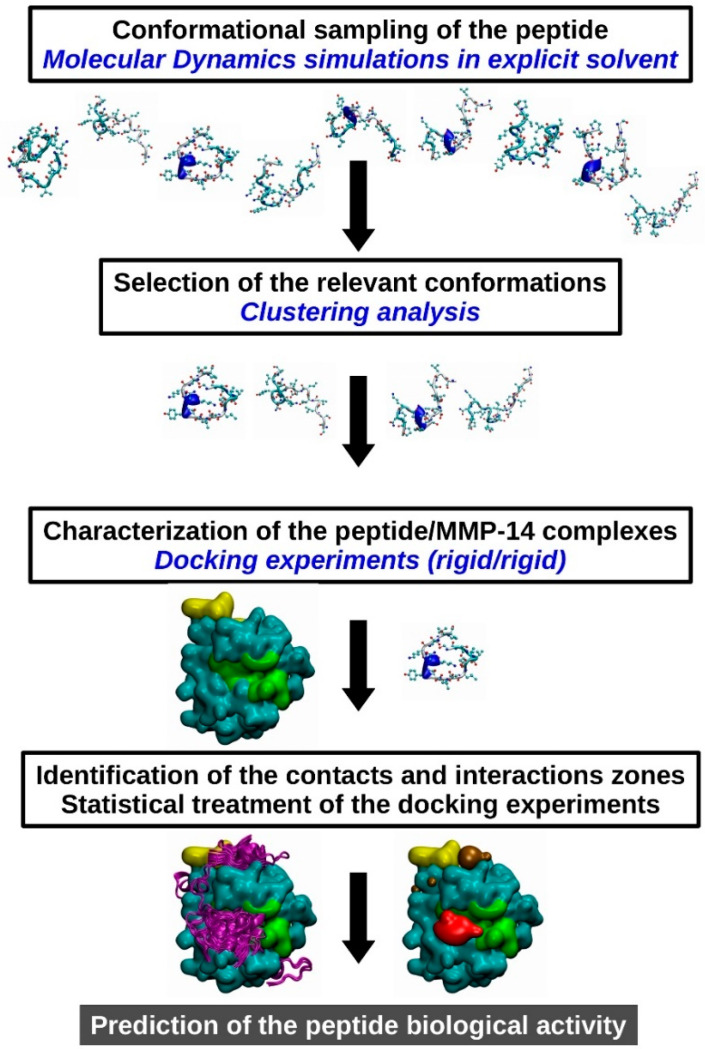
Scheme of the strategy used to decipher and predict the interaction between SLRP-derived peptides and the catalytic domain of MMP-14. The workflow designed for the present study is a 4-step process: (i) the intrinsic flexibility of the peptides is explored through classical MD simulations, (ii) the most relevant conformations are selected using clustering analysis, (iii) peptide/MMP-14 complexes are determined using a rigid/rigid docking software, and finally (iv) contacts and interactions areas are statistically characterized.

Due to the diversity of the size of the considered peptides, ranging from 10 to 17 amino acids, we decided to characterize in silico the interaction between MMP-14 and SLRP-derived peptides through rigid/rigid docking techniques. Indeed, the use of regular ligand/protein docking software, such as Autodock4.2 [51] or Autodock Vina [52], was hindered or could be impaired by the high number of degrees of freedom generated by the backbone and sidechains (in the case of Autodock, the number of degrees of freedom ranging from 44 for D9M or F9M to 72 for lumcorin was much higher than the limit considered as acceptable, e.g., 32). In order to overcome this problem, as illustrated in Figure 2, in the present work, the following sequence of simulation steps were performed:

(i) Molecular dynamics simulations were performed in explicit solvent (see description hereafter) and allowed the conformational sampling of the energy landscape of the considered peptides.
(ii) A clustering analysis was performed with an optimized cut off with which 1 to 5 major conformations could be extracted.
(iii) Docking experiments were performed between the peptide conformations selected in step (ii) and the catalytic domain of MMP-14.
(iv) The SLRP peptides/MMP-14 complexes were analyzed, first to identify the contact and interactions interfaces and secondly to statistically characterize the MMP-14 residues implicated in the formation of the complexes.

Steps (i) and (ii) ensured the indirect consideration of the intrinsic flexibility of the peptides.

Detailed information related to each step is given in the following paragraphs.

### 2.3. Molecular Dynamics Simulations

For each peptide presented in Table 1, molecular dynamics (MD) simulations were performed in order to determine the best conformations to be used for simulations of molecular docking. Simulations were setup with GROMACS [53] and run for 100 ns. The clustering tool of the GROMACS package was used in order to group the 10,000 conformations of each trajectory into different classes, using the gromos algorithm and an adaptative cut off ranging from 2.2 to 2.8 Å. To each class, a percentage of the total population was assigned, and then the number of conformations was chosen to be used in the docking experiments. The flexibility of the peptide was thus indirectly taken into account by considering different conformations in rigid protein/protein docking experiments. The GROMACS simulation package [54,55] was used to perform molecular dynamics simulations focusing on each investigated peptide. The values of the energy parameters associated with the description of atoms and their interactions belonged to the OPLSAA force field [56,57]. Depending on their size, the isolated peptides were placed in boxes with side lengths varying from 40 to 190 Å, ensuring that a given peptide would not interact with its images when the periodic boundary conditions were applied. Prior to the production phase of the simulations, water (TIP3P model [58]) and Cl counter ions were added in the boxes and 5000 steps of energy minimization were performed using the steepest descent algorithm in order to relax the structures. The equilibration of the systems was operated at a temperature of 310 K and for 500 ps in the isothermal-isobaric ensemble. Then, MD simulations were carried out for 100 ns in the NPT ensemble (pressure of 1 bar controlled by the Berendsen algorithm, and temperature of 310 K controlled by the V-rescale algorithm). The SHAKE algorithm [59] was used in order to freeze the length of the bonds involving hydrogen atoms. This also made it possible to integrate the equation from classical mechanics with the Verlet algorithm and an integration step of 2 fs. The Particle Mesh Ewald (PME) algorithm [60,61] was used to compute the coulombic interactions (cut off at 1.8 nm), and the van der Waals interactions were evaluated with a potential-shifting function (shift applied at 1.3 nm and cut off applied at 1.4 nm). 

### 2.4. Rigid Peptide-Protein Docking Experiments

The Hex software [62] was used to perform the docking experiments. Hex was submitted to the CAPRI test, which is used to validate a docking method on known/characterized molecular complexes [63]. The configurable parameters of Hex were tuned based on the re-docking experiment: TIMP-2 was docked on MMP-14 and the solutions were compared to the crystallographic reference (PDB code = 1BQQ [64]) through the evaluation of the RMSD between the crystallographic structure and the best solution provided by the docking experiments. All docking results presented in this paper were obtained with the surface correlation method, the OPLS electrostatic potential, and the (bumps + OPLS) post processing protocol.

### 2.5. Identification of the Contact and Statistical Analysis

The Hex program provided the 100 best solutions according to an energy criterion. Each position of these solutions could be represented by the center of gravity of each peptide. The ensemble of the solutions could then be represented by 100 coordinate triplets corresponding to the positions of the centers of gravity of all the solutions. These triplets were grouped into clusters using the PAM (Partition Around Medoids) method [65]. This algorithm allowed us to associate to each cluster with the different structures of the peptides populating it. Each cluster could be located on the surface of MMP-14 and then the appropriate MMP-14 regions of interest could be identified.

The interactions between MMP-14 and the peptides were studied with the help of the CONTACT program (which is part of the CCP4 suite [66]). Contacts were then identified when a distance between two atoms of different residues was lower than 4 Å. Focusing on a region of interest, contacts with MMP-14 could be compared in a pairwise manner and relevant/interesting residues from this region could be retrieved by statistically comparing the average number of contacts and their deviation. The pairwise comparison was established using a statistical Student’s t-test with a *p*-value lower than 5%.

### 2.6. MMP-14 Activity Assay

The MMP-14 activity was measured in 96-well plates using a 1 μM substrate: 5-FAM/QXLTM520 FRET peptide in the reaction buffer supplied in the SensoLyteR520 MMP-14 Assay Kit (AnaSpec, San Jose, CA, USA). Before all activity assays, MMP-14 was activated using 1mM 4-aminophenylmercuric acetate (APMA) for 2 h at 37 °C. The assays were carried out in triplicate at 37 °C. Fluorescence was measured with a spectrofluorometer (Mithras LB940, Berthold Technologies, Thoiry, France). To determine the direct effect of lumican and decorin on MMP-14 activity in vitro, the recombinant catalytic domain of human MMP-14 (5nM) (amino acids 89-265, Merck Millipore, Nottingham, UK) was pre-incubated at 37 °C overnight before assay with recombinant human glycosylated lumican or glycosylated decorin (100 nM each). Lumican-derived peptides’ effect on the recombinant catalytic domain of human MMP-14 (Merck) was determined by overnight pre-incubation with 100 μM lumican-derived peptides (lumcorin or L9Mc) or their corresponding SCR peptides. The measurement of the hydrolysis of the fluorogenic substrate was monitored after 2 h of incubation at 37 °C. Results were obtained from three independent experiments.

### 2.7. Cell Culture 

Murine B16F1 (CRL-6323^TM^) and human HT-144 (HTB-63) melanoma cells were obtained from ATCC. B16F1 and HT-144 melanoma cells were cultured in standard conditions in DMEM and McCoy’s 5A media, respectively [67]. In all experiments, cell viability was greater than 95%, as assessed by the trypan blue exclusion test.

### 2.8. Migration Assay

Migration assays of B16F1 murine melanoma cells were performed using culture-inserts (ibidi, Biovalley, Marne-la-Vallée, France). Cells were seeded on 24-well plates in culture inserts with 3 × 10^4^ cells per chamber in 70 μL of complete cell culture medium. After 24 h of incubation at 37 °C, the culture inserts were removed, cells were rinsed with Phosphate Buffer Saline (PBS), and the wells were filled with 1 mL of serum-free cell culture medium supplemented with 100 μM lumican-derived peptide (L9Mc) or its corresponding SCR peptides. Cell motility was followed and quantified as previously described [32]. 

For migration assays of HT-144 human melanoma cells, Transwell^®^ polycarbonate membranes (8 μm pore size, 6.5 mm diameter) (Costar, Fisher Scientifique Labosi, Elancourt, France) were used. HT-144 cells and 4 × 10^4^ in 100 μL of McCoy’s 5A were added to the upper chamber. The lower chamber contained 800 μL of medium with 5% FBS. The medium contained mitomycin (5 µg/mL) and either no peptide (control) or 100 μM lumican-derived peptide (L9Mc) or its corresponding SCR peptides. After incubation for 24 h at 37 °C, cells were fixed with ethanol for 20 min and stained with 0.1% crystal violet for 20 min. Cells in the upper chamber were removed by cotton swab. Migrated cells were counted in the entire surface of the filter at 4× magnification under a microscope (EVOS XL core, LifeTechnologies, 91941 Courtaboeuf (Villebon Cedex), France) for every filter (3 filters per condition). Each assay was performed twice.

### 2.9. Proliferation Assay

Cell growth of B16F1 and HT-144 melanoma cells was determined using the MTT test on 96-well plates for 10^4^ cells/well [67]. Cells were grown for 24, 48, and 72 h in the presence of 100 μM L9Mc or its corresponding SCR peptide. Cell growth was then analyzed using 3-[4,5-dimethylthiazol-2-yl]-2,5 diphenyltetrazolium bromide (MTT, Sigma). For this purpose, cells were incubated with culture medium supplemented with 0.5 mg/mL MTT for 3 h at 37 °C. MTT solution was then replaced by DMSO, and absorbance was measured at 560 nm for B16F1 and HT-144 cell proliferation. Each assay was performed twice.

### 2.10. Primary Tumor Growth Analysis

Twelve-week-old wild-type C57BL/6J female mice were subcutaneously injected in the right flank with 10^5^ B16F1 cells in the suspension of 100 μL of Dulbecco’s modified eagle’s medium (DMEM) without or with 200 μg of appropriate peptide (control mice *n* = 10, L9Mc *n* = 10 and L9Mc SCR *n* = 10). Three injections of 100 μL of cell culture medium with 200 μg of appropriate peptide were done at day 6, 9, and 13 in the peri-tumoral area. Mice were evaluated for tumor-sizes at day 6, 9, 12, 13, 14, 15, and 16 from the initial cell injection. On day 16, mice were sacrificed, and tumors were isolated for further analysis. Tumor volume was calculated (in cm^3^) using the formula: volume = a × b^2^ × 0.5, where a is the longest diameter and b the shortest [67].

This study was performed in compliance with The French Animal Welfare Act and following “The French Board for Animal Experiments”. Experiments were conducted under the approval of the French “Ministère de l’Enseignement Supérieur et de la Recherche” (ethics committee C2EA-56) in compliance with the “Directive 2010/63/UE” APAFIS#17470-2018110911091242v2. 

### 2.11. Statistical Analysis of Biological Assays

For proliferation, migration, and MMP-14 activity assays as well as primary tumor growth analysis, the pairwise comparison was established using a statistical student’s *t*-test with a *p*-value lower than 5%.

## 3. Results and Discussion

### 3.1. Molecular Dynamics Simulations

As expected for short peptides, all five investigated SLRP-derived peptides presented a finely tuned dynamic equilibrium between various conformations. Nevertheless, the analysis of the 10,000 extracted snapshots allowed the computation of the average contents in the secondary structure and this analysis gave the following classification from the most structured to the less structured peptide: F9M (25.3%), L9M (24.8%), L9Mc (21.1%), lumcorin (18.5%), and D9M (14.7%). Considering the small discrepancies in the averages, from the local secondary structure point of view, the five peptides can be considered as equivalent.

**Figure 3:**
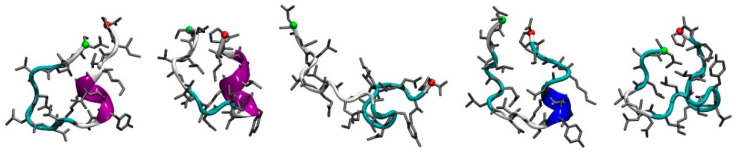
Representative structures of lumcorin peptides. From left to right, the first five conformations of lumcorin, extracted from MD simulations, are presented using cartoon and licorice representations. The green and red spheres represent the first and last alpha carbon of lumcorin, respectively.

As illustrated in Figure 3 for lumcorin peptide, the five first conformations extracted from the MD simulation all present some coil and turn secondary structures. For three of the clusters (first, second, and fourth), some helical secondary structures can be observed. Among the clusters and their representative conformations extracted from the MD simulations, only the ones corresponding to a minimum population threshold were kept for the next step of the workflow, consisting in the docking experiments. As presented in Table 2, for each peptide derived from lumican (lumcorin, L9M and L9Mc), a single conformation was used in the docking experiments, whereas for the peptides derived from decorin (D9M) and fibromodulin (F9M), five and two conformations were used, respectively.

### 3.2. Molecular Docking

**Figure 4:**
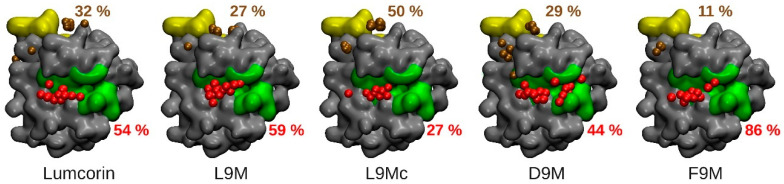
Anchoring of peptides on catalytic and MT-Loop regions. From left to right, localization of lumcorin, L9M, L9Mc, D9M, and F9M peptides on the MMP-14 surface. Two regions of the MMP-14 proteins are prone to interactions: the catalytic region, depicted in green, and the MT-Loop region depicted in yellow. Spheres are used to place the center of mass of the 100 best solutions and the color code indicates their localization at the surface of MMP-14: red spheres are in the catalytic region and spheres in brown are in the vicinity of the MT-Loop. The colored numbers indicate the percentages of solutions associated with each region.

The protocol described previously in the materials and methods section highlights, as depicted on Figure 4, two regions of interest in terms of MMP-14/peptides interaction: the catalytic zone (colored in green in Figure 4) and the MT-Loop (colored in yellow in Figure 4). In addition, on the five panels of Figure 4, the centers of mass of the 100 docked peptides are represented as colored van der Waals spheres with respect to the region they belong to (red for peptides in the vicinity of the catalytic zone and brown in the vicinity of the MT-Loop) and the associated numbers give their respective percentage of the total population. For all the peptides, considering the two highlighted areas of MMP-14, more than 70% of the total number of docking solutions are encompassed. In addition, for all cases, except the L9Mc, the catalytic zone of MMP-14 is the one that is mostly in contact with the peptide, with at least 44% of the docking solutions in the case of D9M’s first conformation and at most 86% of the docking solutions in the case of F9M’s first conformation. The 100 best docking solutions of the L9Mc peptide on MMP-14 stand apart from the 4 other sets of solutions obtained with the other peptides since the MT-Loop is the area of MMP-14 that is mostly in contact with the cyclic peptide.

The total energies associated with the best poses of the SLRP-derived peptides on MMP-14 are presented in Table 3. While lumcorin/MMP-14 and L9M/MMP-14 complexes display similar total energies (−486 and −462 kcal/mol, respectively), D9M/MMP-14 and F9M/MMP-14 complexes display higher total energies (−435 and −372 kcal/mol, respectively), thus reflecting less favorable complexes and correlating the fact that the peptides derived from decorin and fibromodulin are inactive with respect to MMP-14 activity. The total energy of the best pose of the L9Mc/MMP-14 complex (−447 kcal/mol) does not allow us to state with certainty about the inhibitory effect of L9Mc since this value can be considered as similar to the value of L9M or D9M.

### 3.3. Statistical Treatment of the Interaction and Characterization of the Contacts

**Figure 5:**
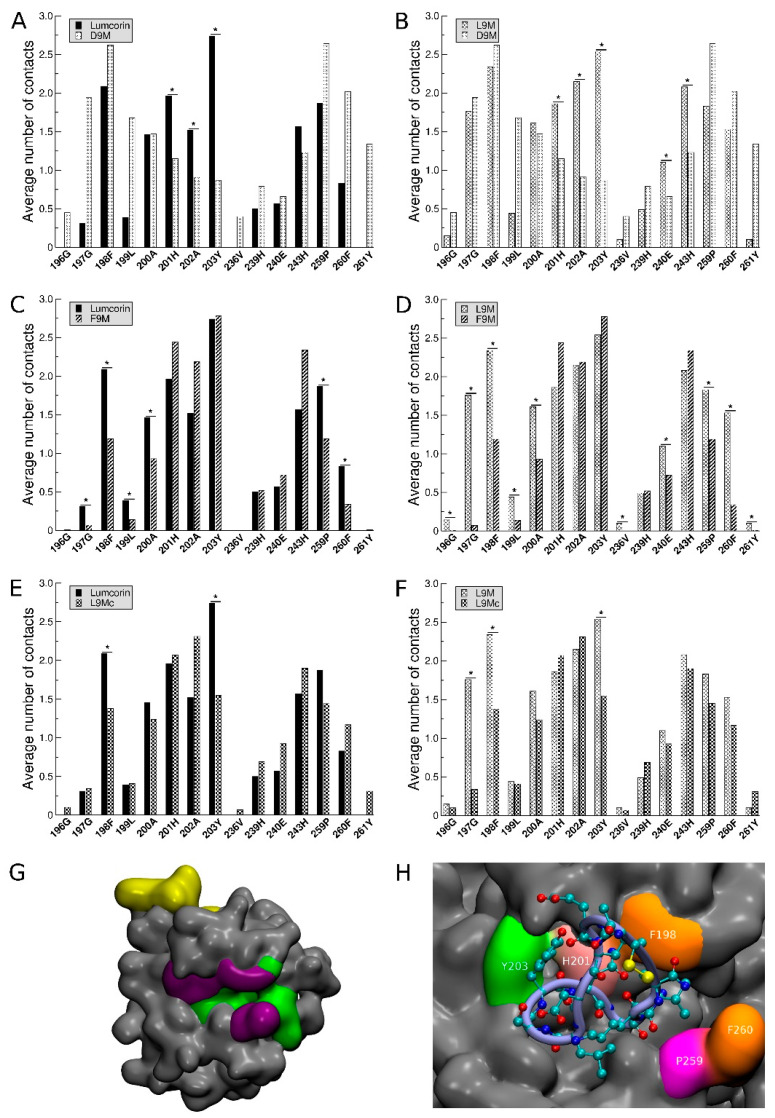
Pairwise comparison of the number of contacts between MMP-14 and different SLRP-derived peptides. The average numbers of contacts (hydrophobic or through hydrogen bonds) and their root mean square deviations computed over the 100 dockings were evaluated for each residue of the MMP-14 catalytic region. Panels (**A**) to (**D**) correspond to the training set of the protocol since inhibitory peptides (lumcorin and L9M) are compared to inactive peptides (D9M and F9M); panels **A**, **B**, **C**, and **D** display the comparison of lumcorin and D9M, L9M and D9M, lumcorin and F9M and L9M, and F9M, respectively. In the case of D9M and F9M, for which there is more than one representative structure (5 for D9M and 2 for F9M) extracted by the clustering analysis, all the conformations were compared to the representative structure of lumcorin and L9M. For clarity purposes, only the comparisons with the first cluster are presented. Panels **E** and **F** display the comparison of L9Mc with lumcorin and L9M, respectively. Error bars (root mean square deviations) are not represented on the different panels but were taken into account for the comparison of the number of contacts with the Student’s *t*-test. Stars identify the residues of MMP-14 displaying significant differences between the considered SLRP-derived peptide. Based on the comparison of the average number of contacts between peptides whose activity is known, key residues of the catalytic zone could be highlighted (panel **G**, violet surface), and docking solutions associated with L9Mc situated in the catalytic zone evidenced contacts involving some of these key residues (panel **H**). *, *p* < 0.05.

Based on the results presented in Figure 4, we decided to thoroughly analyze the docking results corresponding to peptides located in the catalytic zone. For each residue belonging to this zone (196G, 197G, 198F, 199L, 200A, 201H, 202A, 203Y, 236V, 239H, 240E, 243H, 259P, 260F, and 261Y) and over the 100 structures of lower energies obtained from the docking experiments, the average number of interactions (and the associated standard deviation) made between this residue and the peptide was evaluated (see Figure 5). In order to calibrate the methodology, the interactions made by the peptides, whose activity towards MMP-14 was experimentally known, were compared in a pairwise manner. The set of peptides constituted lumcorin, L9M, D9M, and F9M. The average number of contacts was compared, and significant differences were searched for using statistical Student’s *t*-test with a *p*-value lower than 5%. The pairwise analyses between lumcorin, L9M, D9M, and F9M (see Table 4) show that the specific contacts made with inhibitory peptides are not random. Indeed, from the lumcorin/D9M and L9M/D9M comparisons, the contacts made with residues 201H, 202A, and 203Y are highlighted; in parallel, from the lumcorin/F9M and L9M/F9M comparisons, the contacts made with residues 197G, 198F, 259P, and 260F are highlighted. Taken together, the results summarized in Table 4 suggest that the following residues (highlighted in violet on Figure 5G) are crucial in the MMP-14/SLRP-derived peptides interactions: 197G, 198F, 201H, 202A, 203Y, 259P, and 260F.

### 3.4. Predicting the Activity of L9M Cyclic Peptide

L9Mc, whose activity towards MMP-14 was unknown experimentally, was compared to lumcorin and L9M. The significant differences in the average number of contacts were assessed, especially for the seven key residues highlighted and mentioned previously. From panels E and F of Figure 5, among the seven crucial residues identified through our statistical approach, only two non-adjacent residues can be highlighted as showing significant differences in the number of contacts between L9Mc and the inhibitory peptides: 198F and 203Y. Thus, L9Mc was predicted to inhibit MMP-14 activity. Figure 5H illustrates the contacts made by one conformation of L9Mc associated with a docking solution localized in the catalytic zone. It can be observed that five out of the seven key residues of MMP-14 are involved in the interaction with the cyclic peptide.

In order to validate our in silico protocol, it will be necessary to enrich the database of experimental results on which our strategy was built. Other potential and possible improvements could be related to the MD results. In the present study, all the conformations extracted from MD were treated the same way without taking into account the fact that a given conformation could be more or less preponderant than another. In the future, it will be necessary to weight the comparisons of the number of interactions by taking into account the population associated with the conformations. Finally, the predicting protocol was focused on the catalytic region of MMP-14, but the results presented in Figure 4 showed that the MT-Loop region could also be interesting. This could suggest that the inhibitory effects of SLRP-derived peptides could also be related to interactions taking place in this region. As such, it will be interesting to apply the same methodology to the MT-Loop region.

### 3.5. L9M Cyclic Peptide Inhibits MMP-14 Activity

**Figure 6:**
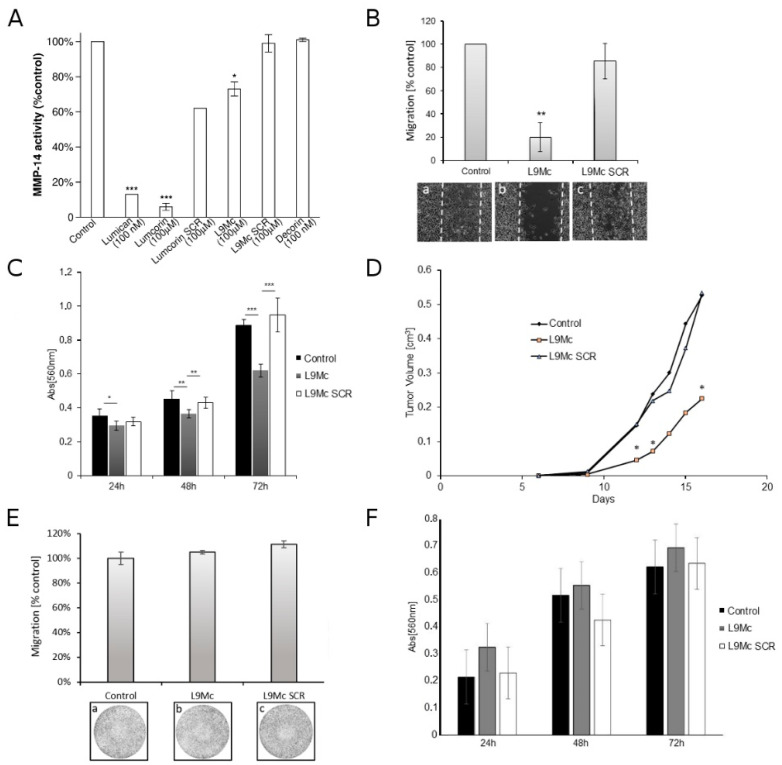
Effect of L9Mc on MMP-14 activity, melanoma cell migration, proliferation, and primary tumor growth. (**A**) MMP-14 assay. Comparison of the effects of lumican, lumcorin and its scrambled (SCR) peptide, L9Mc and its scrambled (SCR) peptide, and decorin on MMP-14 activity measured using the fluorimetric SensoLyte^R^ 520 MMP-14 assay kit as described in the Materials and Methods. Recombinant human MMP-14 was preincubated overnight with 100 nM lumican or 100 μM lumcorin or L9Mc peptide. Data are presented as mean values ± SD from three independent experiments. (**B**) Migration of B16F1 murine melanoma cells in the presence of L9Mc peptide or its control. Cells were plated on a 24-well plate, 3 × 10^4^ cells per chamber of culture-insert. After 24 h of incubation, the culture-inserts were withdrawn, and cell migration was monitored for 48 h by computer-assisted phase contrast videomicroscopy. Representative images of cell positions after 48 h of migration in the absence of peptide (**a**), in the presence of 100 μM L9Mc (**b**), or of its SCR peptide (**c**) are displayed on the bottom panels. Migration was quantified as the percent of recovered area by cells. Graphs represent the mean values ± SD calculated from 4 microscopic fields per insert. The experiment was done in triplicate. (**C**) B16F1 cell proliferation. Cells were grown for 24, 48, and 72 h in the absence (control) or in the presence of 100 μM L9Mc or its SCR peptide. Cell growth was measured by the MTT colorimetric test at 560 nM. Results were reported as means ± SD of sextuplicate values from three independent experiments. (**D**) Tumors were generated by subcutaneous injection of 10^5^ B16F1 melanoma cells in the absence of peptide (control), in the presence of 100 μM L9Mc peptide, or of its scrambled (SCR) peptide. The kinetics of the melanoma tumor volume was measured as described in the Materials and Methods. *: Significant differences in tumor volume between L9Mc peptide-treated mice and control or L9Mc SCR peptide-treated mice are shown. (**E**) Migration of HT-144 human melanoma cells in the presence of L9Mc peptide or its control. Transwell^®^ polycarbonate membranes (8 μm pore size) were used. HT-144 cells and 4 × 10^4^ in 100 μL of McCoy’s 5A were added to the upper chamber. The lower chamber contained 800 μL of medium with 5% FBS. The medium contained mitomycin (5 µg/mL) and either no peptide (control), or 100 μM lumican-derived peptide (L9Mc) or its corresponding SCR peptides. After incubation for 24 h at 37 °C, migrated cells were counted on the entire surface of the filter at 4x magnification under a microscope (EVOS XL core, LifeTechnologies) for every filter (3 filters per condition). Each assay was performed twice. Representative images of cell positions after 24 h of migration in the absence of peptide (**a**), in the presence of 100 μM L9Mc (**b**), or of its SCR peptide (**c**) are displayed on the bottom panels. (**F**) HT-144 cell proliferation. Cells were grown for 24, 48, and 72 h in the absence (control) or in the presence of 100 μM L9Mc or its SCR peptide. Cell growth was measured by the MTT colorimetric test at 560 nM. Results are reported as means ± SD of sextuplicate values from two independent experiments. *, *p* < 0.05; **, *p* < 0.01; ***, *p* < 0.001.

To determine the effect of L9Mc on the activity of the recombinant human catalytic domain of MMP-14, activated MMP-14 was incubated overnight with glycosylated lumican or its derived peptides (lumcorin, L9Mc) or decorin followed by measurement of the enzymatic activity. Lumican decreased MMP-14 activity by 87%, lumcorin by 92%, and L9Mc by 33% (Figure 6A). The L9Mc SCR peptide had no significant effect (Figure 6A). The results of the present report confirm not only that lumican, in contrast to decorin, specifically decreases the MMP-14 activity of the catalytic domain of the enzyme, but also show that lumcorin is more efficient than L9Mc. L9Mc had no effect on MMP-2 and MMP-9 activity (data not shown).

### 3.6. L9M Cyclic Peptide Inhibits the Migration of B16F1 but Not HT-144 Melanoma Cells In Vitro

Lumican was shown previously to inhibit cell migration [67]. Lumcorin was identified and was demonstrated to inhibit melanoma cell chemotaxis [48] and L9M was later shown to mimic the lumcorin effect in vitro [32]. To investigate whether L9Mc altered the migration of B16F1 and HT-144 cells, cell migration assay was performed. In the absence of peptide, as well as in the presence of the SCR L9Mc peptide, B16F1 cells nearly completely closed the wound after 48 h. In contrast, L9Mc (100 μM) decreased B16F1 cell migration by nearly 80% (Figure 6B) as compared to control cells. The corresponding SCR L9Mc peptide had no significant effect on cell motility. These results show that L9Mc, similarly to lumican or lumcorin or L9M [32], is able to decrease the migration of the B16F1 murine melanoma cell line in vitro. In contrast, the migration of human melanoma HT-144 cells was not significantly inhibited by L9Mc peptide (100 μM) as compared to its scrambled or control cells (Figure 6E). Similar results were obtained on a second human melanoma cell line (SK-MEL-28, Figure S1A).

While the inhibitory effect of L9Mc on B16F1 cell migration has been demonstrated, its limited inhibitory effect on MMP-14 activity led us to investigate its role on cell proliferation.

### 3.7. L9M Cyclic Peptide Inhibits the Proliferation of B16F1 but Not HT-144 Melanoma Cells In Vitro

Previous studies described the anti-proliferative property of lumican [67]. Several sequences responsible for the inhibition of melanoma cell proliferation were determined [32,48]. Lumcorin and a shorter 10 amino acid peptide (linear L9M) derived from lumcorin’s central part were shown to mimic the lumican effect in vitro [32]. To investigate whether the cyclic L9M peptide (L9Mc) was able to reproduce lumican’s effect, the proliferation was tested in murine B16F1 melanoma cells, a tumorigenic murine melanoma cell line, and a HT-144 human metastatic cell line. After 24, 48, and 72 h of incubation of the cells with L9Mc, a significant inhibition of cell growth was observed in comparison to its SCR peptide or control without peptide in B16F1 cells (Figure 6C) but not in HT-144 cells (Figure 6F) and SK-MEL-28 (Figure S1B) human melanoma cell lines. After 72 h, the inhibition of B16F1 cell growth reached 40% in the presence of L9Mc peptide (Figure 6C).

### 3.8. Effect of L9M Cyclic Peptide on Melanoma Primary Tumor Developments In Vivo

The study of the in vivo potential inhibitory effects of L9Mc peptide was performed in C57BL/6J female mice, which were subcutaneously injected with a mix of cell suspension of the same genetic background (B16F1 melanoma cells) and L9M cyclic peptide. The tumor progression was monitored for 16 days after cell injection. The tumors were detected 9 days after cell injection. After 16 days from tumor cell injection, a 60% inhibition of tumor growth was observed, in comparison to tumors obtained in the control groups (Figure 6D).

Thus, L9Mc inhibits the proliferation of B16F1 melanoma cells in vitro and in vivo. These results suggest that L9Mc’s inhibitory effect on melanoma tumor progression would be more at the cell proliferation level rather than at the cell invasion level. Indeed, the L9Mc peptide was able to inhibit melanoma cell migration but its significant inhibitory effect on MMP-14 activity was weak. MMP-14 expression has previously been shown to modulate cellular proliferation [68,69]. The fact that our in silico study reveals that L9Mc is more prone to interacting with the MT-Loop over the catalytic pocket might suggest that L9Mc may regulate cell proliferation via MMP-14 and its MT-Loop domain independently of MMP-14 catalytic activity. Interestingly, D’Alessio and collaborators [68] showed that TIMP-2 binding to MMP-14 rapidly activates the extracellular signal-regulated kinase 1/2 (ERK1/2) pathway, which upregulates cell proliferation and migration by a mechanism independent of the proteolytic activity of MMP-14.

This result suggests that L9Mc’s inhibitory effect on melanoma tumor progression would be more at the proliferation level than at the invasion level. Interestingly, in this in silico study, it was shown that the site of interaction between the domains of MMP-14 (catalytic domain or MT-Loop) and lumcorin or L9Mc are different, which might explain, at least in part, the differences between the two peptides in the inhibitory effect of MMP-14 activity. Indeed, their inhibitory effect on MMP-14 appears to be inversely related to the proportion of peptide conformations interacting with the catalytic region. In vivo, L9Mc inhibits tumor development. In vitro, L9Mc inhibits the proliferation as well as the migration of B16F1 cells. These results validate the prediction of the in silico study presented above.

In the present report, L9Mc was shown to be able to inhibit melanoma cell migration. Our group recently reviewed the role of lumican, which is considered as a multivalent effector in wound healing [70]. In addition, our team previously demonstrated that lumican and lumican-derived peptides inhibit breast cancer [39,40] and melanoma cell migration [26,32,48,67,71,72,73], which is mediated through integrin α2β1 [19,28] and MMP-14 interactions [26,30,33].

The results on the B16F1 cell line led us to investigate the effects of L9Mc in cell migration and proliferation of the HT-144 human melanoma cell line, which expressed a relatively high amount of MMP-14 protein (see Figure S2). No significant effect of L9Mc was observed in both functional assays (Figure 6E,F). Altogether, these results suggest that further studies are necessary to better understand the discrepancies of regulation by L9Mc of cell proliferation and migration according to the different cell types and their characteristics. Different hypotheses might be postulated as described in the following part. The difference between species (mice and human cell lines) in the response to L9Mc might be explained by differences in post-translational modifications of MMP-14, such as O-glycosylation [74], in the two cell lines, leading to altered affinities of the peptides to MMP-14 either to its catalytic domain and/or to its MT-Loop domain. As a result, the activity of MMP-14 and cell migration might be differently regulated by L9Mc. In addition, the expression profile of integrin subunits (α2, β1, αv), previously reported by our group as being receptors of lumican [19], was checked in the B16F1, HT-144, SK-MEL-28, and A375 cell lines (Figure S2). All cell lines expressed the αv integrin subunit but not the α2 integrin subunit. Interestingly, a qualitative difference could be observed between B16F1 and HT-144 cells in the expression of the β1 integrin subunit, suggesting a difference in the glycosylation of the β1 integrin subunit between murine and human cells (Figure S2). This glycosylation alteration might interfere in the binding of lumican and its derived peptides to the β1 integrin subunit and indirectly to MMP-14 activity. 

Therefore, lumican peptides might be of interest in pharmacological applications for limiting melanoma progression; however, post-translational modifications of the receptors (integrins) and mediators (MMP-14) should be taken into account. Moreover, the computational approach presented in the present report is rapid and accurate enough to predict the preferential sites of interaction between MMPs and lumican-derived peptides, making it possible to design in silico MMP-14 inhibitors.

Our in silico approach is complementary to the computational approach used by Geistera and collaborators [50] to determine the binding modes of protein-peptide complexes and also to design TGFβRI inhibitors. Based on a small set of experimental data, the protocol elaborated in the present study evaluates the number of contacts made by each residue in the catalytic zone of MMP-14. Although no distinction between hydrophobic nor hydrogen bonding interactions was integrated, the strength of the method was validated since its prediction of L9Mc activity towards MMP-14 was experimentally confirmed in vitro. With reasonable computational cost and effort, our method efficiently performs screening between SLRP peptides of very similar sequences. Once the selection of the relevant peptides has been achieved, the strategy proposed in [50] could then be applied to fully characterize the energetic contribution of the MMP-14 residues in the molecular complex.

## 4. Conclusions

Although similar to the linear L9M sequence, the addition of two cysteines at the N- and C-terminal end of the L9Mc peptide completely changed its conformation and might induce a lesser occupation of the catalytic zone. One of the aims of this study was to model the interactions between MMP-14 and peptides derived from lumican. Key residues were highlighted for the interaction between the inhibitory peptides and the MMP-14 protein. This study established a methodology to identify key residues of the active site of MMP-14. In the future, the experimental data will be enriched thanks to the proposals resulting from our modeling work, and will make it possible to define the interaction model between MMP-14 and its potential peptide inhibitors. Possible and feasible improvements include molecular dynamics results. In fact, for some peptides, several conformations have been used with the same importance for the comparison of the interactions whereas they are more or less predominant. It will be interesting to standardize the comparison results by the percentage of the population of each conformation used so that their weight is taken into account.

## Figures and Tables

**Table 1:** Peptide sequences considered in the study.

Peptide	Sequence	MMP-14 Activity
Lumcorin	SSLVELDLSYNKLKNIP	Inhibitor
L9M	ELDLSYNKLK	Inhibitor
L9Mc	CELDLSYNKLKC	Unknown
D9M	ELHLDNNKLT	No effect
F9M	ELDLSYNQLQ	No effect

**Table 2:** Selection of the conformations for docking experiments.

Peptide	Number of Clusters
Lumcorin	1
L9M	1
L9Mc	1
D9M	5
F9M	2

Number of relevant conformations obtained using the g_cluster module of Gromacs for each of the simulated peptides.

**Table 3:** Comparison of the total energy of the best poses.

Peptide	Total Energy (kcal/mol)
Lumcorin	−486
L9M	−462
L9Mc	−447
D9M	**−435**
F9M	**−372**

Bold numbers correspond to values averaged over the different relevant conformations.

**Table 4:** Summary of the training test.

196	197	198	199	200	201	202	203	236	239	240	243	259	260	261
G	G	F	L	A	H	A	Y	V	H	E	H	P	F	Y
Lumcorin vs. D9M														
					●	●	●							
					●		●							
					●		●							
					●	●	●							
				●		●	●							
L9M vs. D9M														
					●	●	●			●	●			
						●	●	●		●	●			
	●				●		●						●	
				●	●	●	●							
		●		●		●	●							

					✸	✸	✸							
Lumcorin vs. F9M														
	●	●	●	●								●	●	
		●										●	●	
L9M vs. F9M														
●	●	●	●	●				●		●		●	●	●
●	●	●				●		●				●	●	●
	✸	✸										✸	✸	

Comparison of the average number of contacts between known inhibitory peptides (lumcorin and L9M) and known unactive peptides (D9M and F9M). (●) indicates the residues of the MMP-14 catalytic zone for which the number of contacts are significantly higher for the inhibitory peptides. (✸) indicates the residues that are crucial in the interaction between MMP-14 and inhibitory peptides.

## Data Availability

The data presented in this study are available on request from the corresponding author.

## Supplementary Materials

The following are available online at https://www.mdpi.com/article/10.3390/cancers13194930/s1, Figure S1: Effect of L9Mc on SK-MEL-28 melanoma cell migration and proliferation, Figure S2: Profile of expression of integrin subunits (α2, β1, αv) and MMP-14 in the SK-MEL-28, A375, B16F1 and HT144 cell lines analyzed by western immunoblotting.
