# Supplementary material for: Differential MMP-14 Targeting by Lumican-Derived Peptides Unraveled by In Silico Approach
Source: Cancers (Basel). 2021 Sep 30;13(19):4930. doi: 10.3390/cancers13194930 (PMC8507859; doi:10.3390/cancers13194930)
Supplement: Supplementary file 1 [file cancers-13-04930-s001.zip › cancers-1387272-supplementary.pdf]

Supplementary data

Materials and methods:

Cell culture

Murine B16F1 melanoma cells (CRL-6323™) and human A375 (CRL-1619™), SK-MEL-28 (HTB-72™), HT-144 (HTB-63™) melanoma cells were obtained from ATCC. B16F1 and HT-144 melanoma cells were cultured in standard conditions in DMEM and McCoy's 5A medium media, respectively. Human A375 and SK-MEL-28 cell lines were cultured in DMEM medium in standard conditions. In all experiments, cell viability was greater than 95%, as assessed by trypan blue exclusion test.

Migration assay

Migration assays of human SK-MEL-28 cells were performed using culture-inserts (ibidi, Biovalley, Marne-la-Vallée, France). Cells were seeded on 24-well plates in culture-inserts with $3×10^4$ cells per chamber in 70 µL of complete cell culture medium. After 24h of incubation at 37°C, the culture inserts were removed, cells were rinsed with Phosphate Buffer Saline (PBS) and the wells were filled with 1 mL of serum-free cell culture medium supplemented with 100 µM lumican-derived peptide (L9Mc) or its corresponding SCR peptides. Cell motility was followed and quantified as previously described [1]. Each assay was performed twice.

Proliferation assay

Cell growth of SK-MEL-28 melanoma cells was determined using MTT test on 96-well plates for 10^4 cells/well [2]. Cells were grown for 24, 48 and 72h in the presence of 100 µM L9Mc or its corresponding SCR peptide. Cell growth was then analyzed using 3-[4,5-dimethylthiazol-2-yl]-2,5 diphenyltetrazolium bromide (MTT, Sigma). For this purpose, cells were incubated with culture medium supplemented with 0.5 mg/ml MTT for 3h at 37°C. MTT solution was then replaced by DMSO and absorbance was measured at 560 nm. Each assay was performed twice.

Western blot immunoblotting

Protein extract from total cell lysates were prepared as previously described [1]. Total cell proteins (30µg) were subjected to electrophoresis in a 0.1% SDS polyacrylamide gel. Proteins were transferred onto Hybond-P PVDF membranes (GE Healthcare, Orsay, France) by electroblotting and detected using specific antibodies. The following primary antibodies were used: mouse monoclonal anti-human α2 integrin subunit (611016; BD Transduction Laboratories), mouse monoclonal anti-human β1 integrin subunit (AB1952P; Millipore), mouse monoclonal anti-human αv integrin subunit (#60896; Cell signaling), rabbit polyclonal antibody directed against the hinge region of human MMP-14 (ab38971; Abcam), and goat anti-human actin (Santa Cruz Biotechnology, Heidelberg, Germany). The corresponding secondary antibodies conjugated to horseradish peroxidase were purchased from GE Healthcare (Orsay, France). The bands were revealed by the ECL Prime Chemiluminescence Detection reagent (GE Healthcare) as indicated by the manufacturer. The chemiluminescence signal was captured using a ChemiDoc™ MP Imaging System (Bio-Rad) adjusting the exposure time so that all dots were below pixel saturation.

Results

A) Migration and proliferation assays on SK-MEL-28 human melanoma cell line.

The results on HT144 cell line led us to investigate the effects of L9Mc in cell proliferation and migration of SK-MEL-28 human melanoma cell line. No significant effect of L9Mc was observed in functional assays using SK-MEL-28 cell lines (Figure S1).

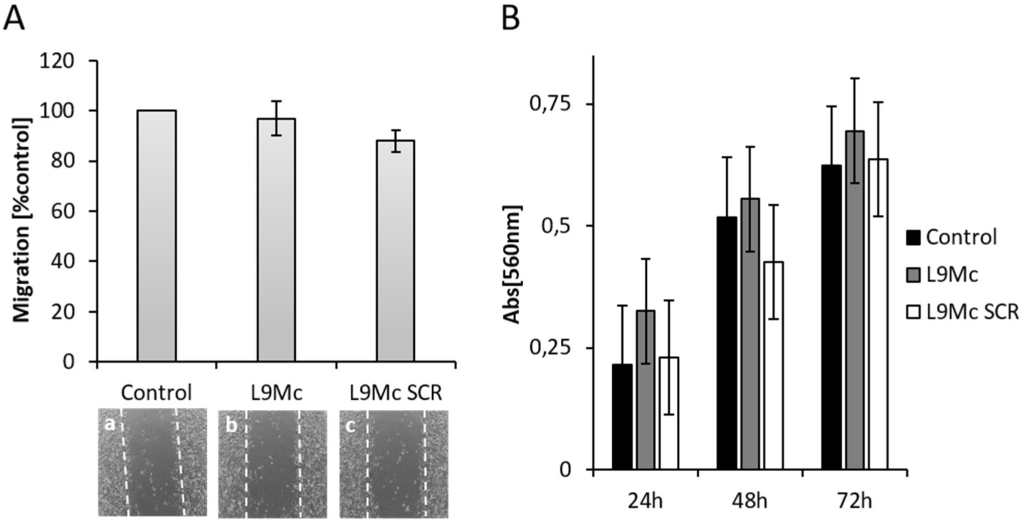

Figure S1. Effect of L9Mc on SK-MEL-28 melanoma cell migration and proliferation. A) Migration of SK-MEL-28 cells in the presence of L9Mc peptide or its control. Cells were plated on 24-well plate, 3×10^4 cells per chamber of culture-insert. After 24h of incubation, the culture-inserts were withdrawn and cell migration was monitored for 48h by computer-assisted phase contrast videomicroscopy. Representative images of cell positions after 48h of migration in the absence of peptide (a), in presence of 100 μM L9Mc (b) or of its SCR peptide (c) are displayed on bottom panels. Migration was quantified as percent of recovered area by cells. Graphs represent the mean values ± SD calculated from 4 microscopic fields per insert. The experiment was done in duplicate. B) SK-MEL-28 cell proliferation. Cells were grown for 24, 48 and 72h in absence (control) or in presence of 100 μM L9Mc or its SCR peptide. Cell growth was measured by MTT colorimetric test at 560 nM. Results were reported as means ± SD of sextuplicate values from two independent experiments.

B) Profile of expression of integrin subunits (α2, β1, αv) and MMP-14 in the SK-MEL-28, A375, B16F1 and HT144 cell lines.

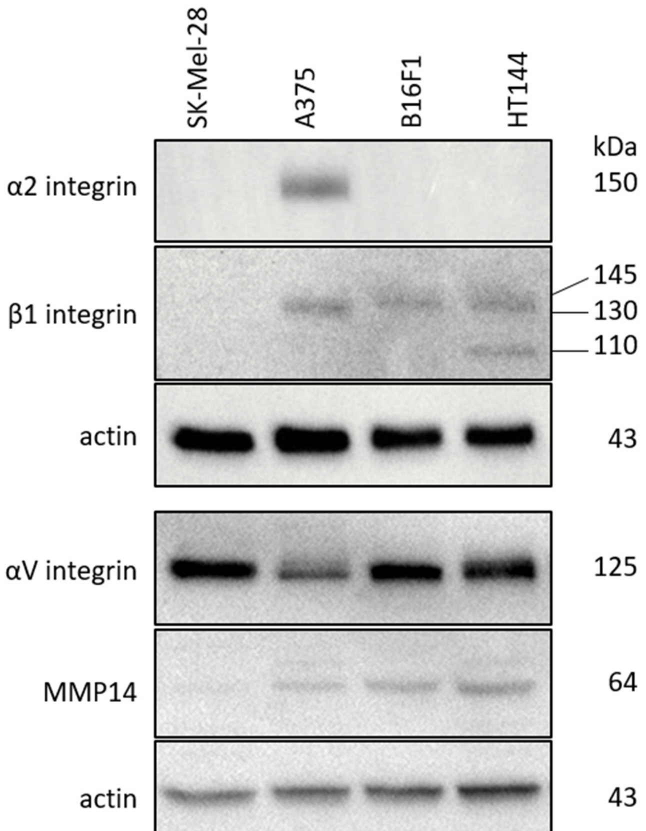

Figure S2. Profile of expression of integrin subunits (α2, β1, αv) and MMP-14 in the SK-MEL-28, A375, B16F1 and HT144 cell lines analyzed by western immunoblotting. All cell lines expressed αv integrin subunit. Except A375 cells, none of

them expressed α2 integrin subunit. Interestingly, a qualitative difference could be observed between B16F1 and HT144 cells in the expression of β1 integrin subunit. Two bands (145 and 110 kDa) could be detected in HT144 cells in contrast to a unique band (145 kDa) observed in B16F1 cells suggesting a difference in the glycosylation of the β1 integrin subunit between murine and human cells. This glycosylation alteration might interfere in the binding of lumican and its derived peptides to β1 integrin subunit and indirectly to MMP-14 activity. In parallel, the relative amount of expression of MMP-14 is displayed for each cell line.

References

1. Pietraszek, K.; Brézillon, S.; Perreau, C.; Malicka-Blaszkiewicz, M.; Maquart, F.X.; Wegrowski, Y. Lumican-derived peptides inhibit melanoma cell growth and migration. *PLoS One.* **2013**, *8*, e76232.
2. Vuillermoz, B.; Khoruzhenko, A.; D'Onofrio, M.F.; Ramont, L.; Venteo, L.; Perreau, C.; Antonicelli, F.; Maquart, F.X.; Wegrowski, Y. The small leucine-rich proteoglycan lumican inhibits melanoma progression. *Exp Cell Res.* **2004**, *296*, 294–306.
